# TpUB05, a Homologue of the Immunodominant *Plasmodium falciparum* Protein UB05, Is a Marker of Protective Immune Responses in Cattle Experimentally Vaccinated against East Coast Fever

**DOI:** 10.1371/journal.pone.0128040

**Published:** 2015-06-08

**Authors:** Jerome Nyhalah Dinga, Mark Wamalwa, Dieudonné Lemuh Njimoh, Moses N. Njahira, Appolinaire Djikeng, Rob Skilton, Vincent Pryde Kehdingha Titanji, Roger Pellé

**Affiliations:** 1 Biotechnology Unit, Faculty of Science, University of Buea, Buea, Cameroon; 2 Biosciences eastern and central Africa-International Livestock Research Institute (BecA-ILRI) Hub, Nairobi, Kenya; 3 International Livestock Research Institute, Nairobi, Kenya; University of Chile, CHILE

## Abstract

**Introduction:**

East Coast fever, a devastating disease of cattle, can be controlled partially by vaccination with live *T*. *parva* sporozoites. The antigens responsible for conferring immunity are not fully characterized. Recently it was shown that the *P*. *falciparum* immunodominant protein UB05 is highly conserved in *T*. *parva*, the causative agent of East Coast fever. The aim of the present investigation was to determine the role of the homologue TpUB05 in protective immunity to East Coast fever.

**Methods:**

The cloning, sequencing and expression of TpUB05 were done according to standard protocols. Bioinformatics analysis of *TpUB05* gene was carried out using algorithms found in the public domain. Polyclonal antiserum against recombinant TpUB05 were raised in rabbits and used for further analysis by Western blotting, ELISA, immunolocalization and *in vitro* infection neutralization assay. The ability of recombinant TpUB05 (r-TpUB05) to stimulate bovine PBMCs *ex-vivo* to produce IFN-γ or to proliferate was tested using ELISpot and [3H]-thymidine incorporation assays, respectively.

**Results:**

All the 20 cattle immunised by the infection and treatment method (ITM) developed significantly higher levels of TpUB05 specific antibodies (*p*<0.0001) compared to the non-vaccinated ones. Similarly, r-TpUB05 highly stimulated bovine PMBCs from 8/12 (67%) of ITM-immunized cattle tested to produce IFN-γ and proliferate (*p*< 0.029) as compared to the 04 naїve cattle included as controls. Polyclonal TpUB05 antiserum raised against r-TpUB05 also marginally inhibited infection (*p* < 0.046) of bovine PBMCs by *T*. *parva* sporozoites. In further experiments RT-PCR showed that the *TpUB05* gene is expressed by the parasite. This was confirmed by immunolocalization studies which revealed TpUB05 expression by schizonts and piroplasms. Bioinformatics analysis also revealed that this antigen possesses two transmembrane domains, a N-glycosylation site and several O-glycosylation sites.

**Conclusion:**

It was concluded that TpUB05 is a potential marker of protective immunity in ECF worth investigating further.

## Introduction

East Coast fever (ECF), a tick-borne disease caused by *Theileria parva* results in severe economic loss to livestock production, and kills 1 million animals each year [1]. The disease is present in 11 countries in eastern, central and southern Africa [2] and is spreading due to a wider distribution of the tick vector and uncontrolled livestock movement [3]. A well established, safe and affordable vaccine that can confer full and long lasting protection against all parasite populations is still awaited. A live parasite-based vaccine, which is used to immunize the animals by an infection and treatment method (ITM), is available [4, 5] but has serious disadvantages. Its laborious production in addition to the requirement of a liquid nitrogen cold chain for delivery and oxytetracycline co-treatment makes it expensive, whereas animals vaccinated by the ITM protocol remain life-long carriers of the parasite, which poses risk for spread of the disease [6].

Current control methods for East Coast fever include the use of acaricides and immunization by the ITM [7]. The strong immune response engendered by using the ITM vaccine indicates the feasibility of a successful recombinant vaccine against ECF. This implies there are parasite antigens that can induce protection, which could be included in the development of subunit vaccines [8]. Recent advances made in the development of a subunit vaccine against *T*. *parva* based on the p67 sporozoite surface antigen, with induction of immunity to ECF in 50% of vaccinated cattle, shows that more protective antigens need to be identified [9] as it has been suggested for malaria [10].

A study on the apicomplexan parasite that causes malaria in humans, *Plasmodium falciparum*, identified an immunodominant antigen, UB05 (GenBank Accession Number DQ235690; [11]), which was shown to be a potential marker of protective immunity to malaria [11, 12].

The principle of the conservation of gene function suggests that most orthologous gene products play similar roles in closely related organisms [13]. Conceivably, *T*. *parva* orthologue of UB05 could play a similar role in immunity to ECF. The aim of the present investigation was to clone the *T*. *parva* homologue of UB05 designated as TpUB05 (GenBank accession number: KF875450) and to investigate its role in the host-parasite interplay in ECF.

## Materials and Methods

### Ethical Statement

The study reported here was carried out in strict accordance with the recommendations in the standard operating procedures of the International Livestock Research Institute Institutional Animal Care and Use Committee (ILRI IACUC). The ILRI' Experimental Animal Request Form and Protocol for blood collection and polyclonal antibody production was approved by the ILRI IACUC (IACUC ref no. 2013.05).

### Molecular Biology reagents and bacterial strains

The plasmid vectors pGEM-T Easy (Promega) and pET32a+ (Novagen) were used for cloning and protein over-expression. *Escherichia coli* strains JM109 (Promega) and Rosetta (DE3)pLysS (Novagen) were used for bacterial overexpresson. Ni-NTA agarose was from Qiagen GmbH. Restriction enzymes and HisProbe-HRP were purchased from Thermo Fisher Scientific, Inc. (USA). The reagents for PCR amplification, IPTG, agarose and reagents for protein purification were purchased from Applied Biosciences (USA) and Sigma (USA). Oligonucleotides were purchased from Bioneer Corporation (Korea). Animals used in the study were raised at the ILRI Farm Tables 1, 2 and 3.

**Table 1:** Demographic information of cows used for bovine ELISA.

No.	Animal Number	Date of birth	Sex	Breed	Immune status	Day of serum collected post-challenge
1.	BD46	17/03/08	M	Friesian/Ayshire	ITM immunized (IM)	35
2.	BD52	25/01/2008	M	Friesian	IM	35
3.	BD53	15/03/2008	M	Friesian	IM	35
4.	BD54	03/04/2008	M	Friesian	IM	35
5.	BD58	15/03/2008	M	Friesian	IM	35
6.	BD61	10/04/2008	M	Friesian	IM	35
7.	BD63	02/04/2008	M	Gurnsey	IM	35
8.	BD69	03/02/2008	M	Friesian	IM	35
9.	BD71	02/03/2008	M	Friesian	IM	35
10.	BD72	19/03/2008	M	Friesian	IM	35
11.	BD74	16/01/2008	M	Friesian	IM	35
12.	BD76	10/03/2008	M	Friesian	IM	35
13.	BD80	12/03/2008	M	Friesian	IM	35
14.	BD81	15/02/2008	M	Friesian	IM	35
15.	BD88	01/03/2008	M	Friesian	IM	35
16.	BC143	28/08/2007	M	Friesian	IM	52
17.	BC148	02/09/2007	M	Ayshire/Gurnsey	IM	55
18.	BC150	21/09/2007	M	Friesian	IM	52
19.	BC152	19/10/2007	M	Friesian	IM	52
20.	BC161	31/08/2007	M	Friesian	IM	55
21.	BD028	01/12/2007	M	Friesian	Un immunized (infected but not treated) (UIM)	42
22.	BD040	28/11/2007	M	Friesian	UIM	14
23.	BD073	15/02/2008	M	Friesian	UIM	49
24.	BC106A	27/09/2007	M	Ayshire	UIM	14
25.	BC110A	30/08/2007	M	Ayshire/Gurnsey	UIM	14
26.	BC136	20/08/2007	M	Ayshire	UIM	14
27.	BC138	18/09/2007	M	Friesian	UIM	14

**Table 2:** Demographic information of cows used for bovine ELISpot and T-cell Proliferation assays.

No.	Animal Number	Date of birth	Sex	Breed	MHC Class 1 *(BoLa type*)	Immune status	Day of challenge infection post-Immunization
1.	BG033	01/08/2011	M	Friesian	T2a (A10)	ITM immunized	35
2.	BG042	12/10/2011	M	Friesian	A10/12	“	35
3.	BH047	02/12/2011	M	Friesian	A11, A15	“	35
4.	BH051	02/03/2012	M	Ayshire	A12, A14/15	“	35
5.	BH053	29/02/2012	M	Ayshire	A12, A14/15	“	35
6.	BH054	05/11/2011	M	Friesian	A10, A14/15, A18v	“	35
7.	BH055	19/11/2011	M	Ayshire	A11, A15	“	35
8.	BH056	15/11/2011	M	Friesian	A15, A18v	“	35
9.	BB007	23/01/2006	M	Friesian/Ayshire	HD6 (A18)	“	35
10.	BA219	25/02/2005	M	Friesian/Ayshire	HD6 (A18)	“	35
11.	BF091	21/03/2010	M	Friesian	HD6 (A18)	“	35
12.	BF092	05/01/2010	M	Friesian	HD6 (A18)	“	35
13.	BJ58	09/11/2012	M	Friesian	A18	Naїve	0
14.	BJ59	02/02/2013	M	Friesian	A18	Naїve	0

**Table 3:** Background information on rabbits used for polyclonal antibody production against r-TpUB05.

Rabbit Tattoo no.	Date of Birth	SIRE	DAM
911	3/04/2013	New Zealand	Dutch X Chinchilla
912	3/04/2013	New Zealand	Dutch X Chinchilla

### Construction of overexpressing *Theileria parva* TpUB05 clone

Total RNA isolated from *in vitro* culture of the *T*. *parva-*infected bovine lymphocyte cell line F100 TpM 3087 [14] was used to generate a *TpUB05* cDNA clone. Briefly, RT-PCR was performed with 5 μg of total RNA in 50 μl of reaction using the OneStep RT-PCR (Qiagen) Kit as recommended by the manufacturer. The TpUB05 ORF 5’ primer sequence was 5’-GGGATCCGCAGATCTCACCAAACGCAAAC-3’ and the 3’primer was 5’-CAAGCTTTTAGTTTGATCTCCTGAAAC-3’. The first 7 nucleotides being extrinsic to the TpUB05 gene, introducing the underlined unique BamHI and HindIII sites at the 5’ and 3’ ends of the amplified DNA fragment, respectively, and creating the deletion of the ATG initiation codon while conserving the stop codon. The PCR product was purified from agarose gel and cloned into pGEM-T Easy vector using JM109 (Promega). The cloned fragment was excised with BamHI and HindIII from the recombinant plasmid and directionally subcloned into pET32a+ expression plasmid vector. The pET32a+ expression vector possesses six consecutive histidine residues (6xHis tag) for efficient purification of recombinant proteins. The resulting recombinant plasmid was sequenced to verify the *TpUB05* sequence and then transformed into *E*. *coli* Rosetta (DE3)pLysS for overexpression and affinity purification.

Tp5 (GenBank Acc. No. XP_765334), a *T*. *parva* CD8^+^ T-cell antigen encoded by a gene containing 4 exons, was used as an internal positive control as well for positive amplification (it should be noted that Tp5 is a different protein from TpUB05, which is studied in this paper). Tp5 forward fusion primer 5’-CGTATCGCCTCCCTCGCGCCATCAGACGAGTGCGTGTATGCTCGGTAATGGCAG-3’ with the Tp5 specific sequenced underlined and reverse primer 5’-TTATAAATCATCGATATCGAAATC-3’ were used to amplify Tp5 DNA from the third to the fourth exons for 35 PCR cycles.

### Detection of TpUB05 in *T*. *parva* life stages

Expression of the *TpUB05* gene was examined in three developmental stages (sporozoites, schizont, piroplasms) of *T*. *parva* isolate 3087, by RNA amplification using RT-PCR as described above, except that the annealing temperature was 58°C and the PCR was for only 25 cycles. GAPDH (GenBank accession no XM_758925) and p104 (GenBank accession no M29954) were used as positive controls. PCR was performed using gene-specific primers: GAPDH, 5’ primer (5’-ccaactgcctagctccactc-3’) and 3’ primer (5’-catcgacaaagtccgaggat-3’); p104, 5’ primer (5’-TTTAAGGAACCTGACGTGACTGC-3’) and 3’ primer (5’-TAAGATGCCGACTATTAATGACACC-3’). Amplicons of 411bp and 496bp were generated, respectively. They (10 μl) were analyzed by electrophoresis in ethidium-bromide-stained 1.8% agarose gel as described [15].

### Cattle sera

The cow antisera used in this study were from animals of the Friesian and Ayshire breed Tables 1 and 2 reared in the ILRI experimental farm.

### Production of polyclonal anti-sera in rabbits

All animal experimentation procedures were reviewed and approved by the International Livestock Research Institute (ILRI) Institutional Animal Care and Use Committee. Two New Zealand rabbits Table 3 were used for the production of polyclonal antibodies against recombinant TpUB05. The rabbits were fed on pellets (Unga Feeds Limited) and water *ad libitum*. Polyclonal antibody production was done as earlier described [16] with some modifications. Briefly, rabbits were inoculated intramuscularly with 75 μg of recombinant TpUB05 antigen. Rabbits were boosted on days 14 and 28 by intramuscular injection with the same amount of antigen in Titermax adjuvant (Sigma-Aldrich). After the antibody titre reached 1:1000 as tested by ELISA, the final boost of neat antigen was given intraveneously. Then rabbits were sacrificed and blood was collected and used to prepare polyclonal antibodies. The blood was incubated at room temperature for 60 min to allow clotting and then centrifuged at 1500 x g for 10min. The serum supernatant was transferred into a fresh tube and heated in a water bath at 56°C for 60 min to inactivate serum complement that may affect neutralizing antibody assays. Inactivated serum was mixed briefly and subjected to ammonium sulfate precipitation. In ammonium sulfate precipitation, saturated ammonium sulfate was prepared by dissolving 1000 gram ammonium sulfate in 1000ml of water (distilled). This was added slowly to the serum sample while stirring, to a final saturation of 50%, for 30 minutes. The homogenate was span at 4000 rpm for 30 minutes. The pellet was resuspended in ammonium sulfate to 50%, original volume, and span at 4000 rpm/30mins. The supernatant was discarded and the pellet resuspended in PBS as required (small volume). The polyclonal antibody preparation was dialysed extensively three times in PBS. Aliquots were stored at—20°C until use.

### Overexpression and purification of recombinant TpUB05 protein

Overexpression and purification of recombinant TpUB05 (r-TpUB05) was performed as earlier described [17] with the following modifications. A colony of Rosetta (DE3)pLysS strain transformed with TpUB05/pET32a+ plasmid was grown overnight at 37°C in 50 ml LB supplemented with 100 μg/ml Carbenicillin and 34 μg/ml chloramphenicol. The following day, 500 ml LB containing the respective antibiotics was inoculated with the overnight culture and let to grow to an OD of 1.0 at 600 nm. IPTG was then added to a final concentration of 2 mM. Culture was allowed to proceed for 4 hours and harvested by centrifugation in a Beckmann Table Top centrifuge at 2100 x *g* for 10 min. The pellet was resuspended in 40 ml of denaturing lysis buffer (8M urea, 0.1 M NaH_2_PO_4,_ 20 mM Tris-HCl, 30 mM imidazole, pH 8.0, 0.01 M phenylmethylsulfonyl fluoride (PMSF)). Cells were disrupted by sonication (60W, 70% pulser, 15 min). Insoluble bacterial components were removed by centrifugation (2100 x *g*, 10 min). Batch purification was carried out by mixing the clarified supernatant with 1.5 ml of equilibrated Ni-NTA resin slurry for 30 min on a rotator (Denley Spiramix 5). The bound resins were collected by centrifugation (400 x *g*, 5 min) and washed five times with 20 ml Wash buffer (40 mM Imidazole, 100 mM NaH_2_PO_4,_ 300 mM NaCl, 10% glycerol, 0.05% NaN_3_, 0.1% Triton-X-100). The recombinant protein was eluted with 0.5 M imidazole in PBS. The concentration and purity of the protein was measured using a spectrophotometer. 50 mM of L-arginine, 50 mM L-glutamic acid and 0.1 M PMSF were added for solubility and stability to the purified protein samples and dialyzed against PBS and stored at—20°C until use.

### SDS-PAGE and Western blot analyses

Recombinant TpUB05 and *T*. *parva* Muguga parasite total lysate (purified parasite lyzed in PBS by sonication) were analysed by sodium dodecyl sulphate- polyacrylamide gel electrophoresis (SDS-PAGE; 15% plyacrylamide) and Western blotting, as previously described [17].

### ELISA for the antibody responses to TpUB05

The presence of antibodies to TpUB05 in bovine serum was determined by the enzyme linked immunosorbent assay (ELISA) as decribed [17] with modifications. Microtitre plates (Polysorp Nunc, Roskilde, Denmark) were coated with 150 μμl per well of optimized concentration of 10 μg/ml antigen in PBS at 37°C, 2 hours. They were washed thrice with Wash buffer (0.05% Tween 20, 0.25% BSA, 0.02% NaN_3,_ in PBS) followed by blocking with 0.2% casein in PBS containing 0.05% Tween 20. The blocking buffer was removed and antisera added at 1:200 dilution in Wash buffer containing 1% skimmed milk. It was incubated for 30 min at 37°C in an Insel incubator/shaker (Insel Instruments # 1S89). Plates were then washed three times with Wash buffer. 150 μl of anti-bovine IgG-HRP conjugate (Sigma) diluted at 1: 20,000 in Wash buffer containing 1% skimmed milk was added to each well and incubated for 30 min at 37°C with gentle shaking and then washed as above. The reaction was revealed using the substrate 2,2’-azino-di-[3-ethyl-benzothiazoline-6 sulfonic acid] diammonium salt (ABTS). In addition to the positive (Polymorphic Immunodominant Molecule) and negative controls, the fusion partner (Tag only) of the expression vector was also included as another negative control. Optical densities were read at 405nm on a microplate reader (Labsystems Multiskan MCC 340, Helsinki, Finland). A serum is considered positive if the mean OD_405_ of the test wells is greater than the baseline; the baseline being mean control OD_405_ plus three standard deviation (mean control OD_405_ + 3 SD).

### Isolation of bovine PBMC, CD4^+^ and CD8^+^ T cells

Blood (30 ml) was collected from the jugular vein into a syringe containing an equal volume of Alsever’s solution and mixed gently. Then the blood was layered onto Ficoll Paque solution (Pharmacia LKB, Sweden) at a ratio of 3:2 and centrifuged at 900 x *g* for 30 min at room temperature without brakes. Using a sterile pipette the PBMCs were aspirated from the interface, transfered to a sterile tube and topped up with PBS solution. This was centrifuged at 500 x *g* for 10 min at room temperature. The pelleted PBMCs were washed three times in Alsever’s solution by centrifugation at 500 x *g* at room temperature, to remove platelets. 5 ml of Red Blood Cell lysis solution per 10 ml of original blood volume was added to the pelleted PBMCs and left for 5 min at room temperature. 25 ml of PBS was added and the solution centrifuged at 300 x g for 15 min to pellet the PBMCs.

CD4+ and CD8+ T cells were purified from peripheral blood mononuclear cells by magnetic cell sorting as described previously [18]. Briefly, CD4+ or CD8+ cells were labeled with MACS Microbeads carrying either anti-CD4 antibody or anti-CD8 antibody, respectively. The sample was then placed in a MACS column and then in a MACS Separator. The flow-through fraction was collected as the negative fraction depleted of the labeled cells. The column is removed from the separator and the retained cells are eluted as the enriched, positively selected cell fraction.

### Bovine ELISpot assay

The enzyme-linked immunospot (ELISpot) assay was performed with isolated PBMCs, purified CD4+ T-cells or purified CD8+ T-cells using a bovine ELISpot assay kit (MABTECH, ELISPOT) and according to the manufacturer’s instructions. Briefly, plates were coated overnight with the coating antibody provided. 250,000 PBMCs/well and TpUB05 at 10 μg/ml were added to each well on the plates and incubated for 20 hours at 37°C, 5% CO_2_. The plate was washed and the biotinylated detection antibody added, followed by streptavidin-HRP conjugate. The spots were revealed using 3, 3’, 5, 5’-Tetramethylbenzidine (TMB) substrate. Concanavalin A (Con A) was used as the positive control antigen at 5 μg/ml and no antigen was added in the negative control wells. The developed spots were counted using an ELISpot reader. A reaction was considered positive if the stimulation index (SI) was greater than two. The SI was obtained by dividing the mean test spot counts by the mean negative control spot counts.

### T-cell proliferation assay

Isolated PBMCs were adjusted to 2.5 x10^6^ cells/ml of complete RPMI. 100 μl of the cell suspension was added into each well in triplicates in a 96-well plate (Corning, Costar). To each well was added 100 μl complete RPMI containing TpUB05 antigen at 10 μg/ml (test wells), Con A at 5 μg/ml (positive control wells) or no antigen (for negative control wells). The plate was incubated at 37°C in a humified incubator with 5% CO_2_. The cells were pulsed with [3H]-thymidine on day 4. The plate was incubated again overnight for isotope uptake. Using a Harvester, cells were harvested and the filter matts dried and read on a counter. A reaction was considered positive if its stimulation index (SI) was greater than two.

### Immunolocalization assay

The generated anti-TpUB05 antiserum was used to determine which of the stages of *T parva* Mugaga (piroplasm, schizont and sporozoite) did express TpUB05. The cells were spun unto a glass slide using Cytospin (Cytospin 2, Shandon Southern Products Ltd., Cheshire, U.K.), air-dried, and fixed in 4% paraformaldehyde for 30 min at room temperature. The fixed cells were permeabilized using 0.5% Triton X-100 in PBS for 15 min at room temperature. Free sites were blocked with blocking solution (3% BSA, 10% fetal calf serum, in TTBS (0.3 M NaCl, 20 mM Tris·Cl. pH8.0, 0.1% Tween 20. 0.01% NaN_3_)) for 30 min at room temperature. Antisera (neat or at 1:100 dilution) or monoclonal antibody (1:100 dilution) were added to the slide and incubated for 2 hours at room temperature. Washing was done three times for 15 min each, in TTBS. Secondary antibody ((anti-rabbit FITC (Sigma, F-0382) or anti-mouse FITC (Sigma, F-0257)) was added at room temperature for 2 hours in the dark and washed twice with TTBS. Hoechst staining (1:200) was performed for 30 min at room temperature and washed once with TTBS for 5 min. Mounting oil (10mg ρ-phenylenediamine, 1ml PBS, 9ml glycerol) was added to the slide and covered with a cover slip. Nail polish was used to seal cover slip. Slides were observed under a fluorescent microscope (Axio Imager D1 Upright Microscope). Negative control consisted of staining schizonts with pre-immunization serum while positive control involved the staining of schizonts with monoclonal anti-schizont antibody.

### *In vitro* neutralization of *T*. *parva* sporozoites infection

The antibody neutralization of sporozoite infection tests were performed following some modifications of the method earlier described [19]. Briefly, 5 x 10^4^ sporozoites in RPMI 1640 medium with 10% fetal bovine serum and 1:2 dilution of anti-TpUB05 antisera were mixed in each well of a 24-well microtiter plate. After a 30 min incubation at 37°C, 5 x 10^6^ bovine PBMCs from two ITM-immunized and two naive cattle that were randomly selected, were added to each well and the plates were kept at 37°C for 72 hours. Giemsa-stained cytospin smears prepared from each well were examined for the presence of schizonts. Four hundred cells were counted and the percentage bearing schizonts was determined.

### Bioinformatics analyses

TpUB05 recombinant gene construct was sequenced by pyrosequencing using the 454 GS FLX Sequencer (Roche) and analyzed with VecSreen (National Centre for Biotechnology Information; NCBI) to remove vector sequences. The TpUB05 sequence was then analysed with BLAST of the GenBank non-redundant nucleotide and protein databases (NCBI) using the family of algorithms with default parameters [20]. The open reading frame was determined by open reading frame (ORF)-Finder and conserved-domain search was performed using NCBI conserved domain search [21]. The significant match with TP04_0076 was then selected for further analyses. Predictions of the physicochemical and structural properties of the derived protein sequence were performed using the protein analysis software available at Expasy.

### B-cell and T-cell epitope prediction

The antibody epitopes predictions were made on 30^th^ November 2014 using the IEDB analysis resource Consensus tool [22–24].

Prediction of T-cell epitopes on TpUB05 that binds MHC Class I haplotypes was done using NetMHCplan version 2.8 on the 30^th^ November 2014 [25]. Strong binding peptides have a rank threshold below 0.5 while weak binding peptides have a rank threshold between 0.6 and 2.0.

### Statistical analysis

The non parametric analysis of variance (ANOVA), Mann-Whitney, Kruskal-Wallis and Friedman tests performed for the evaluation of fixed effects on different traits by using SAS software, Release 8.2 (SAS Institute Inc., Cary, NC) and SPSS Statistics 17.0 Release 17.0.0 (Aug, 23 2008) respectively. A 95% confidence limit was used. A *p*-value less than 0.05 was considered statistically significant.

## Results

### Molecular Cloning and expression of TpUB05

**Fig 1:**
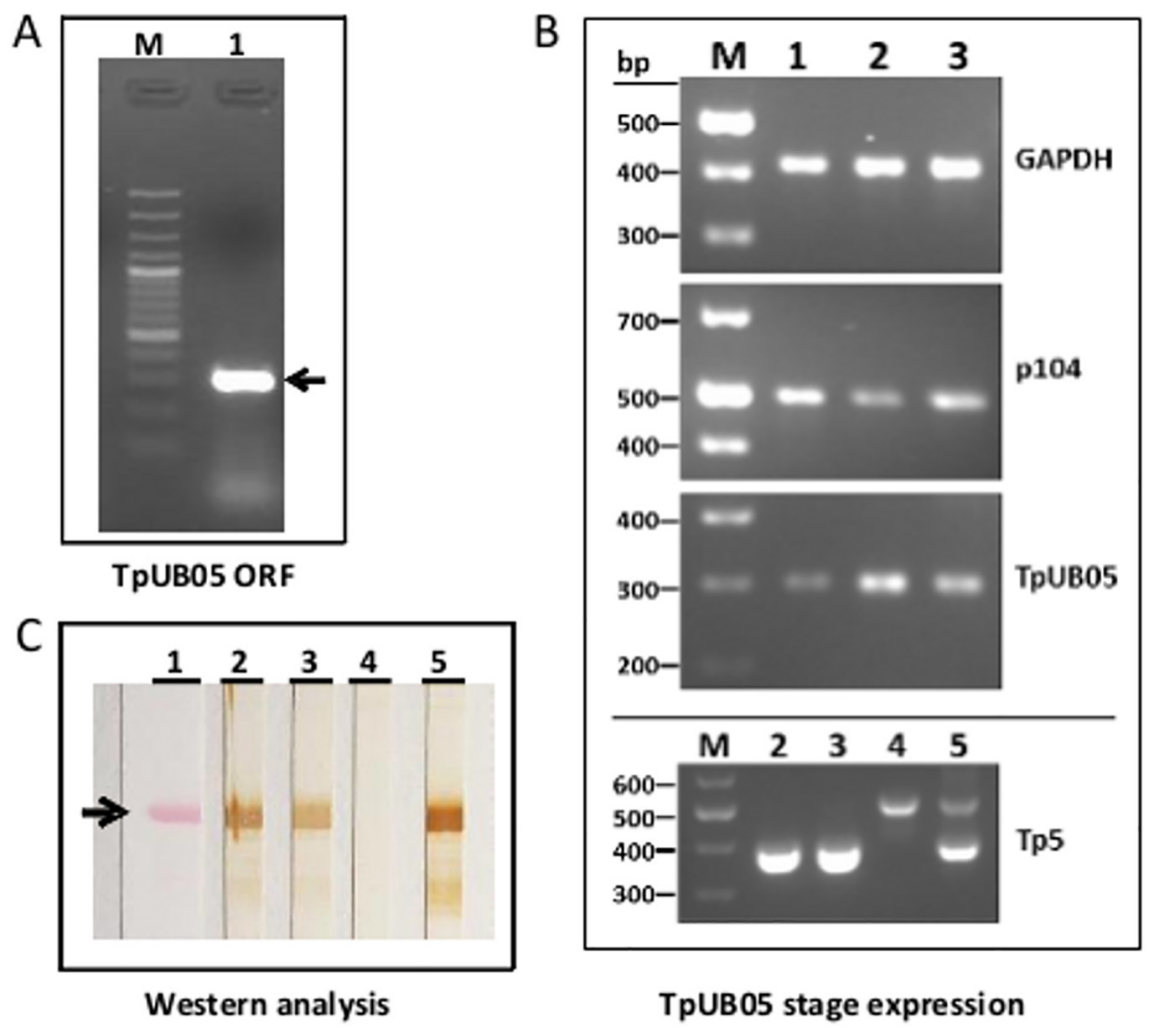
Cloning, expression and immunoanalysis of TpUB05. 1A: TpUB05 full-length ORF generated by RT-PCR amplification of schizont RNA from *T*. *parva*-infected lymphocyte cell culture using specific forward and reverse primers. Lane M, 100bp size markers (Fermentas) and lane 1, TpUB05 amplicon (indicated with an arrow). 1B: RT-PCR analysis of stage expression. Samples: 10μl amplicon per lane of 1.8% agarose gel. Genes: TpUB05, 291 bp; p104, 496 bp; GAPDH, 411 bp. Lanes: M = 100bp plus ladder (Fermentas); lane 1: sporozoites; lane 2: schizonts; lane 3: piroplasms. The amplicons of Tp5 were 385 bp from RNA-derived cDNA (lanes 2 and 3) and 505 bp from genomic DNA (lane 4) due to the presence of a 120 bp-long intron. When cDNA was spiked with 25 ηg of genomic DNA, two bands of 385 and 505 bp were amplified (lane 5). 1C: Western blot analysis of recombinant TpUB05 protein. Purified recombinant TpUB05 protein was run on SDS-PAGE and transferred onto nitrocellulose paper. Strips were then probed with antisera. Lane 1: Ponseau S stain; lane 2: anti-TpUB05 antiserum; lane 3: antiserum from ITM-immunized cattle BD053; lane 4: antiserum from un-immunized infected but not treated cattle BD040; lane 5: anti-UB05 antisera.

Using *TpUB05* specific primers, a 291 bp DNA fragment encoding a 96 amino acid long protein of 11kDa was amplified from schizont derived RNA Fig 1A. Subsequent cloning and expression in pET32a+ vector yielded a 28 KDa recombinant fusion protein as shown by SDS-PAGE. The increase in size was fully accounted for by the presence of 6xHis tag, a S Tag domain and a 109 amino acid thioredoxin fusion protein partner.

### Expression and immunolocalization of TpUB05

Semi-quantitative PCR experiments demonstrated that TpUB05 was transcribed in all the developmental stages of *T*. *parva* examined. When compared with the house keeping gene GAPDH, the relative abundance of *TpUB05* RNA increases as the sporozoite form of the parasites differentiate into the main pathogenic stage of ECF, the schizonts Fig 1B. In addition, the TpUB05 expression profile differed from that of the control antigen p104, that show the highest and lowest levels of transcription in the sporozoite and schizont forms of the parasite, respectively.

**Fig 2:**
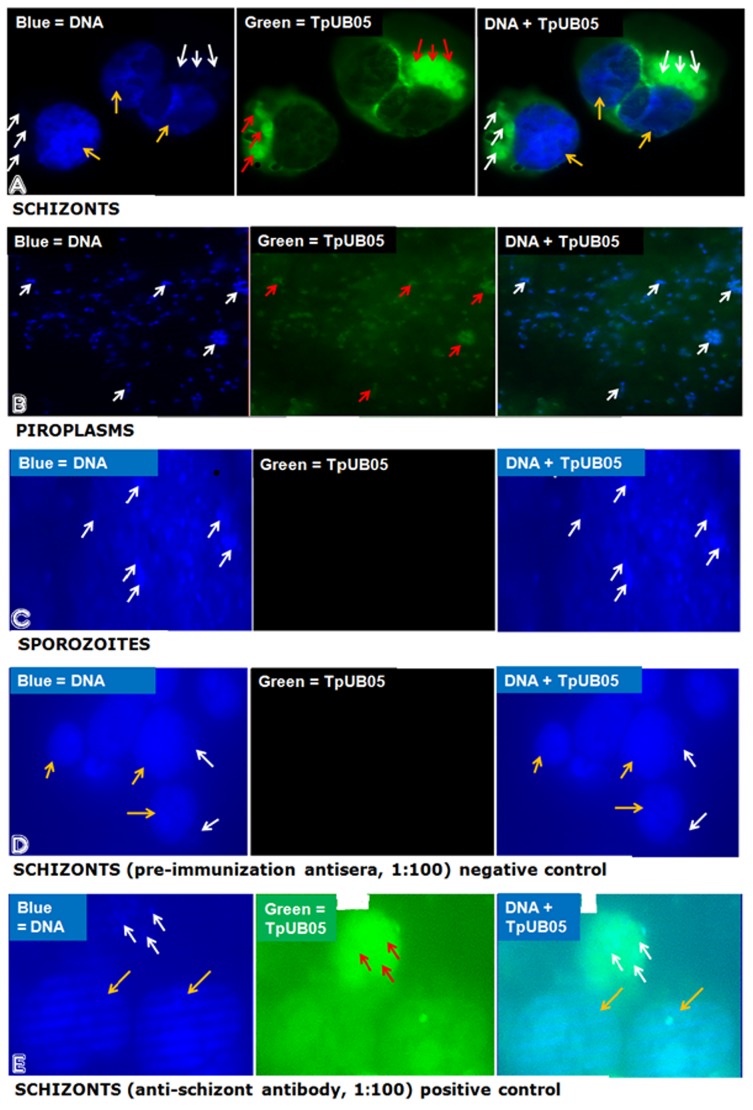
Immunolocalization staining of the various stages of *T*. *parva* Muguga strain using different antibodies /antisera. A: staining of piroplasms using anti-TpUB05 antisera. B: staining of schizonts using anti-TpUB05 antisera. The sporozoites showed no expression of TpUB05. Negative controls (staining of schizonts with pre-immunization) showed no reaction while the Positive control (staining of schizonts using monoclonal anti-schizont antibody) gave a strong reaction. For all panels, white arrow indicates selected parasite DNA (blue), yellow arrow indicates selected bovine PBMC DNA and red arrow indicates TpUB05 protein (green).

To examine the spatial distribution and determine what parasite stage(s) expressed TpUB05, fixed parasites and *T*. *parva*-infected cells were stained with antibodies against TpUB05. The antibodies specifically stained the schizonts inside the infected cells, with no preferred localizsation in any specific parasite compartment Fig 2A. The antibodies also detected TpUB05 in the piroplasms of *T*. *parva* Muguga Fig 2B but the intensity of the stain was stronger in the schizonts than in the piroplasms. However, there was no obvious expression of TpUB05 by the sporozoites under the conditions employed. The positive control also showed a strong reaction. This result is consistent with the transcription data that showed high, medium and low TpUB05 transcript abundances in the schizonts, piroplasms and sporozoites, respectively Fig 1B.

### Western blot analysis of recombinant TpUB05 protein

To determine the antibody responses to recombinant TpUB05 (r-TpUB05) western blot analyses were conducted with sera from ITM-immunized and control cattle. It can be seen from Fig 1C, lane 2 that the r-TpUB05 protein was recognized by rabbit anti-r-TpUB05 antiserum. Antiserum from an ITM-immunized cattle also recognized r-TpUB05 Fig 1C, lane 3. But antiserum from an un-immunized cattle (infected but not treated) did not recognize r-TpUB05 Fig 1C, lane 4. r-TpUB05 was also recognized by anti-UB05 antiserum Fig 1C, lane 5.

### Antibody responses to TpUB05

**Fig 3:**
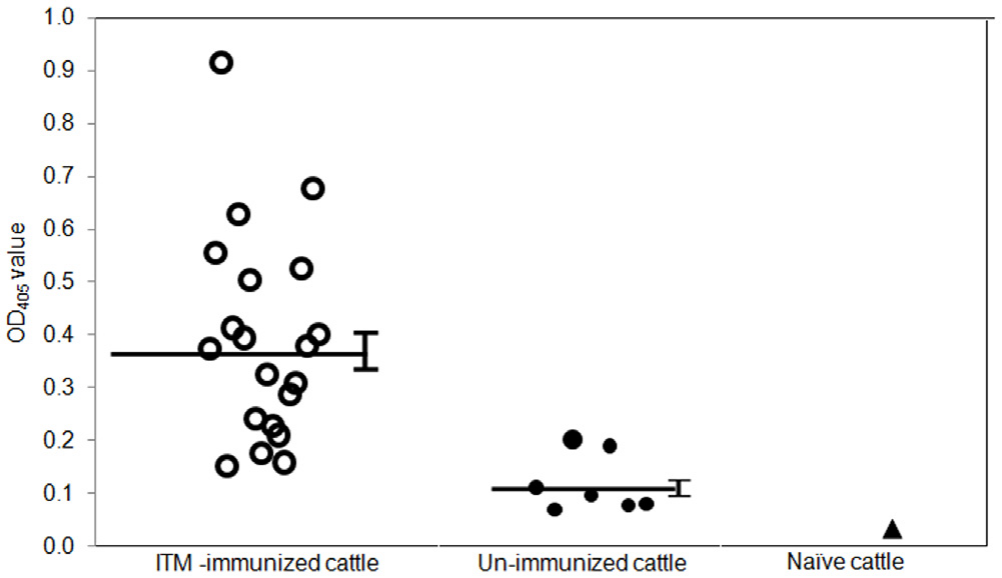
ELISA. **Recognition pattern of TpUB05 by antisera from ITM-immunized and un-immunized (infected but not treated) cattle**. Sera from twenty eight animals were used in ELISA to detect the presence of specific antibody to TpUB05. Twenty four of the 28 cattle studied clearly posses specific antibody to TpUB05 while the ITM immunized cattle possess a significantly higher amount of the antibody compared to the un-immunized cattle and the naïve cattle (*p*<0.0001). The vertical capped bar indicates the standard deviation of each study group. An antisera is considered positive if the mean test OD_405_ is greater than the baseline (mean control OD + 3 standard deviation). Experiment run thrice in duplicates.

Using ELISA, the immunogenicity of antibodies from ITM-immunized cattle against TpUB05 was compared with that of sera from infected/but not treated animals. An antiserum was considered positive if the mean OD_405_ of the test wells was greater than the baseline (baseline = mean control OD_405_ + 3 Standard Deviations). The result showed that 25 of the 28 (89.3%) cattle studied possess antibodies against TpUB05. All the twenty ITM-immunized cattle recognized TpUB05 and produced significantly higher anti-TpUB05 antibody titers compared to the infected/but not treated cattle (*p*<*0*.*0001*) Fig 3. The pre-immunisation serum from one naїve cattle included in this study did not have antibodies against TpUB05 and served as the negative control. The fusion partner (Tag only) gave OD values that were comparable to the naїve serum (data not shown). This study strongly indicated that TpUB05 is an immunogenic antigen in cattle and probably induced specific antibodies in the ITM vaccinated cattle.

### PBMCs from immunized animals recognized TpUB05

**Fig 4:**
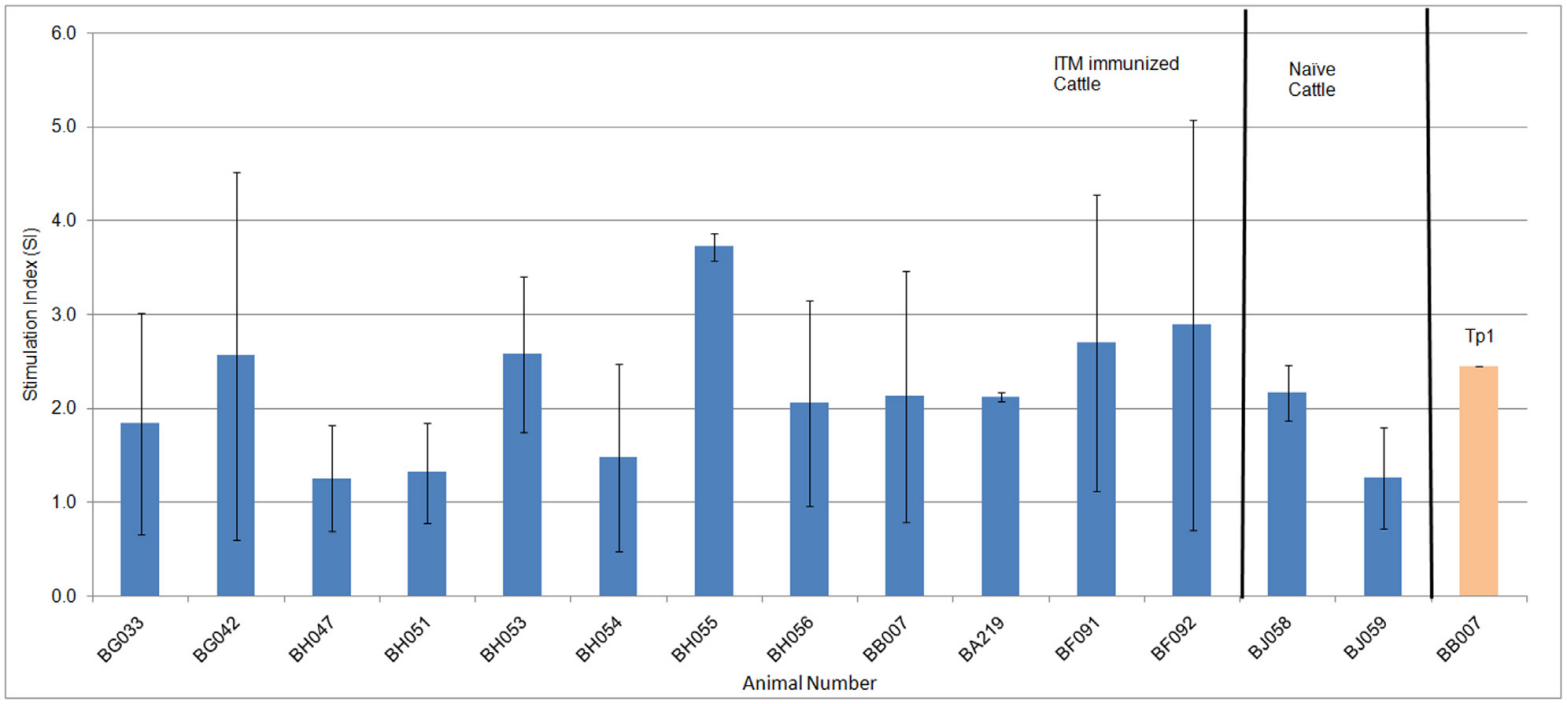
ELISpot assay. Using bovine ELISpot assay, PBMCs from twelve ITM immunized cattle and two naïve cattle were stimulated with TpUB05 for the production of interferon-Gamma. The SI of the ITM immunized cattle were significantly higher than those of the naive cattle (*p*<0.0332). Eight out of the 12 ITM immunized cattle recognized TpUB05 and produce IFN-γ. One of the two naïve cattle also recognized TpUB05. The well-characterized potential subunit vaccine candidate was included in the assay as an internal positive control. An animal is considered positive if two conditions are met; (i) sample SI is greater than the mean+3SD of the control, (ii) the SI of the sample is at least twice that of the control. Experiment was run twice in triplicates.

Having probed the antibody response in the section 3.4 above, the next series of experiments was designed to probe the T-cell response to TpUB05. The T-cell response to TpUB05 was determined using ELISpot assay. Isolated PBMCs from cattle were stimulated *ex-vivo* with r-TpUB05 for the production of interferon-gamma (IFN-γ) as measured by bovine ELISpot assay. Eight out of the twelve (67%) ITM-immunized animals of different major histocompatibility complex (MHC) class I genotypes tested produced IFN-γ in a manner significantly higher than the naїve animals studied (*p* < 0.0332) Fig 4. Most responders were cattle expressing MHC class I of the alleles A18 (5 animals), A15 (3 animals) and A12 (2 animals). However, one of the two naive cattle was shown to release significant amounts of IFN-γ. CD8+ T cell target antigen (Tp1) which is well characterizsed to be the target of CD8 T-cell responses of *T*. *parva* immune cattle and which is restricted by the MHC class I allele A18 was included in the study as an internal positive control Fig 4, Tp1 yellow bar.

**Fig 5:**
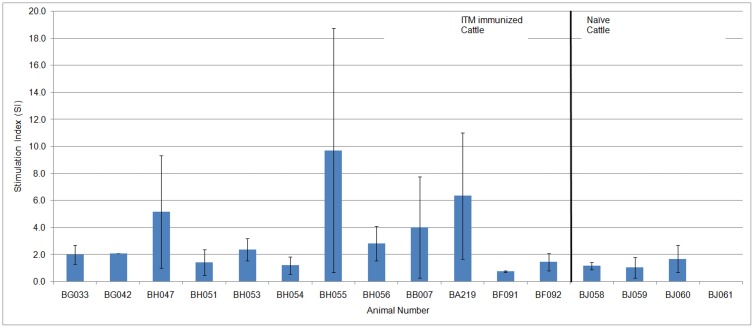
T-cell proliferation assay. Proliferation assay was used to measure the ability of TpUB05 to cause the proliferation of bovine PBMCs by [3H]-thymidine incorporation. PBMCs from 16 cattle was used in this assay; 12 ITM-immunized and 4 naïve cattle. The ITM-immunized cattle’s PBMCs proliferated more than that of the naïve cattle (*p* < 0.029). PBMCs from 7 out of the 12 ITM immunized cattle recognized TpUB05 and proliferated significantly. None of the naïve cattle’s PBMCs proliferated due to the presence of TpUB05. The naïve cattle BJ061 has an average SI of 0.03. An SI>2 is considered as positive. The experiment was run thrice in triplicates.

T-cell proliferation induced *ex-vivo* by r-TpUB05 was also measured by [3H]-thymidine incorporation. The PBMCs from ITM-immunized cattle proliferated significantly (*p* <0.029) as compared to the naїve cattle with mean SI values of 3.28 and 0.98, respectively. TpUB05-induced proliferation of PBMCs occurred in eight out of the twelve ITM immunized cattle taking an SI cut-off point of 2 Fig 5.

**Fig 6:**
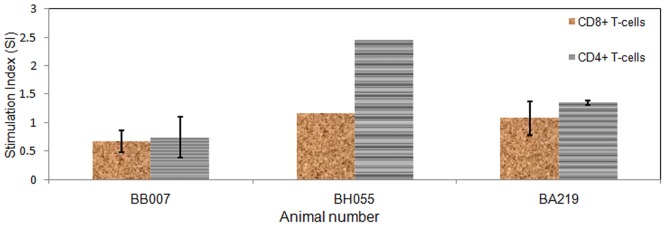
ELISpot assay to assess subset of PBMCs stimulated by TpUB05 to produce interferon-gamma. CD4+ and CD8+ T-cells were separated from three (3) animals and stimulated with r-TpUB05 using bovine ELISpot assay. The CD4+ subpopulations of BH055 and BA219 appear to be significantly stimulated by TpUB05 (*p* = 0.013) compared to their CD8+ subpopulations. The CD4+ T-cells of BB007 did not significantly produce a higher amount of IFN-γ compared to its CD8+ T-cells subset. Comparing the response of all three animals tested, CD4+ T-cells produced a higher amount of IFN-γ (*p* = 0.046) compared to the CD8+ T-cells. An SI>2 is considered positive. The experiment was run in triplicates.

When the different subsets of T-cells (CD4+ and CD8+) were sorted from three animals (BB007, BH055, BA219) and stimulated with r-TpUB05, CD4+ cells of BH055 and B219 produced IFN-γ in a manner that was significantly higher (*p* = 0.013) compared to their CD8+ T-cells. The CD4+ T-cells of BB007 did not significantly produce IFN-γ than its CD8+ T-cells. Comparing all tested animals, CD4+ T-cells produced a significantly higher amount of IFN-γ (*p* = 0.046) Fig 6 using Friedman test.

### Infection inhibition assays using anti-TpUB05 antibodies

**Fig 7:**
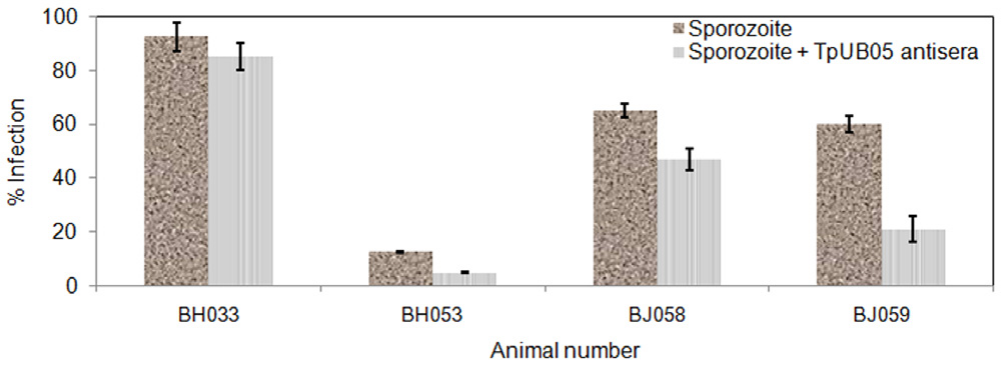
Neutralization of sporozoite infectivity assay. Polyclonal antisera raised against r-TpUB05 was used in a neutralization of *in vitro* infectivity assay to test its ability to block the infection of bovine PBMCs by *T*. *parva* parasite. PBMCs from 4 animals were used. This assay shows that anti-TpUB05 antisera blocked the infection of bovine peripheral mononuclear cells by *T*. *parva* Muguga sporozoites from 3 animals; BH053, BH058, and BJ059. There was also blocking of infection in BH033 but this was not significant. The experiment was run twice in triplicates.

We used the neutralization of *in vitro* infectivity assay to test the ability of polyclonal anti-TpUB05 antiserum to block the infection of bovine PBMCs by *T*. *parva* Muguga sporozoites 3087. The efficiency of the sporozoite infectivity varied among the 4 animals used. The lowest being with BH053 and the highest was observed with BH033 Fig 7. Antibody against TpUB05 was able to reduce by 30%, 50% and 70% the sporozoites infection of PBMCs from the cattle BH058, BH053 and BJ059, respectively Fig 7. In contrast, the anti-TpUB05 antiserum did not significantly block the infection of PBMCs from cattle BH033 Fig 7. The reason for this inactivity was not explored further. Generally speaking, the blocking of PBMCs infection by anti-TpUB05 antiserum was marginally significant (*p* < 0.046) using Friedman test.

### Bioinformatics characterization of TpUB05

**Fig 8:**
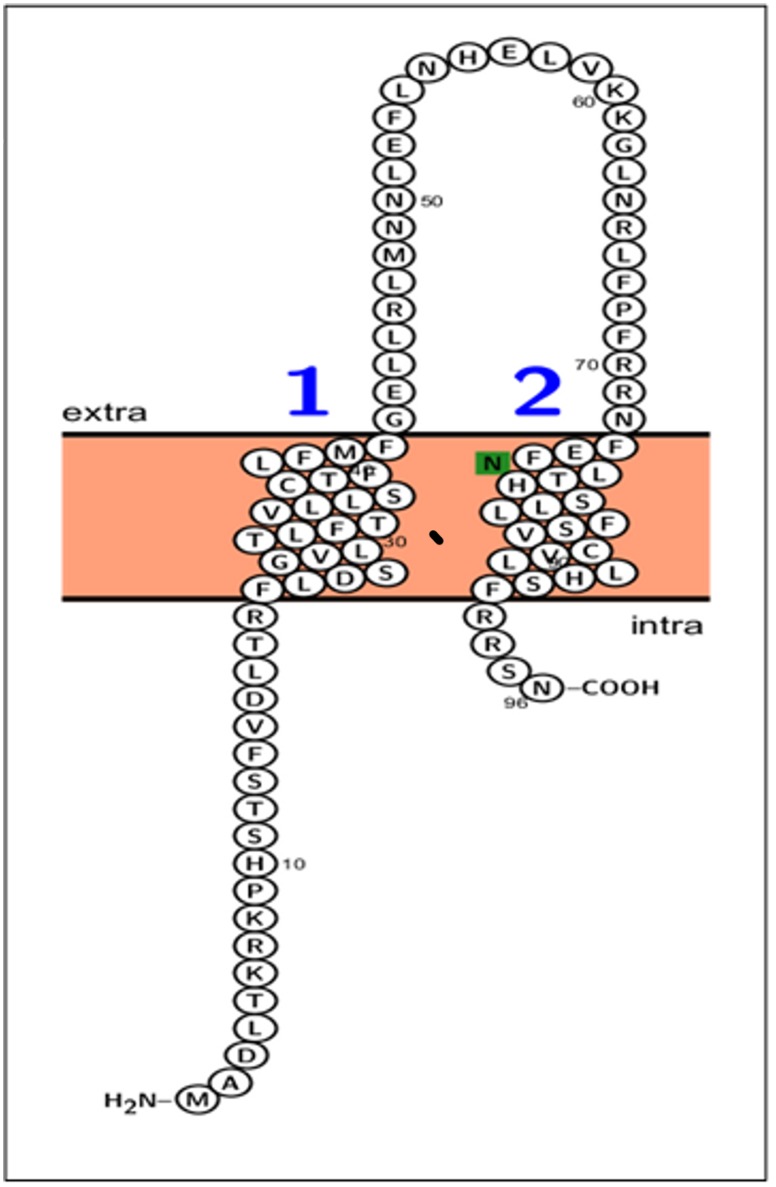
Schematic representation of TpUB05 across *T*. *parva* membrane using Protter version 1.0. Extra: extracellular domain; intra: intracellular domain. 1 and 2 denote the two transmembrane domains whereas the unique N-glycosylation aa residue is in green color.

Bioinformatics analysis did not identify a signal peptide in the TpUB05 antigen. The software Protter version 1.0 [26] predicted two transmembrane domains spanning amino acid residues 20–41 and 73–92 that flanked a hypothetical surface exposed domain Fig 8 and lead to the exposure of both the N- and the C-terminals of TpUB05 in the cytosol. The unique N-glycosylation consensus sequence Asn-Leu-Thr at residues 76–78 was localized in the second transmembrane domain. TpUB05 has 8 and 7 serine and threonine residues, respectively, which constitute potential O-glycosylation sites. None of the identified glycosylation sites was found in the predicted surface exposed domain (aa 42–72).

**Fig 9:**
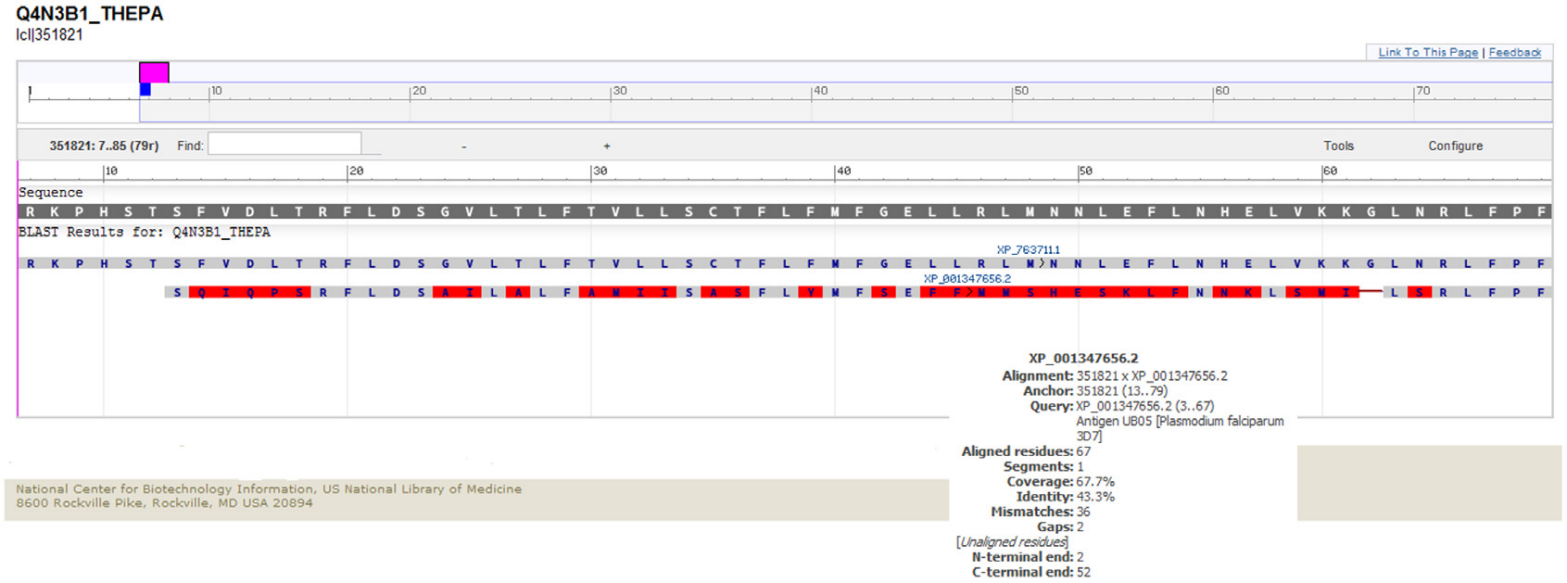
Pairwise alignment of TpUB05 (XP_763711.1) and antigen UB05 (XP_001347656.2) using NCBI BLAST. The *in silico* analysis shows that there is 43.3% identity between the two sequence and 67% similarity.

BLAST analysis indicated that antigen UB05 has 43.3% identity and 67% similarity with TpUB05 Fig 9. *In silico* analysis using prediction tools predicted five antibody epitopes in TpUB05 that span nearly the whole sequence Table 4. Algorithms that predict T-cell epitopes indicated the presence of these epitopes in TpUB05 that can bind strongly to most of the common BoLA types in the world Table 5.

**Table 4:** Prediction of antibody epitopes on TpUB05 using the algorithm developed by Hoof *et al*., 2009 found at www.iedb.org.

No.	Amino acid sequence	Peptide position on antigen	Length (residues)	Score
1.	THSLLFSVCVLLHSF	78->92	15	1.256
2.	DSGVLTLFTVLLSCTFLFMFGELLRLM	22->48	27	1.187
3.	FLNHELVKKG	53->62	10	1.101
4.	STSFVDLTRF	11->20	10	1.089
5.	NRLFPF	64->69	6	1.056

**Table 5:** Prediction of T-cell epitopes that can bind the most prevalent BoLA types.

No.	BoLA type	Peptide sequence	Rank Threshold
1.	BoLA-HD6	RLFPFRRNFEF	0.10
2.	”	RLFPFRRNF	0.17
3.	BoLA-D18.4	RLFPFRRNFEF	0.12
4.	”	RLFPFRRNF	0.25
5.	”	FMFGELLRLM	0.40
6.	”	FEFNLTHSL	0.40
7.	BoLA-1*00901	RLFPFRRNFEF	0.08
8.	”	FMFGELLRLM	0.15
9.	”	RLMNNLEF	0.25
10.	”	FEFNLTHSLLF	0.30
11.	”	RLFPFRRNF	0.30
12.	”	FMFGELLRL	0.50
13.	BoLA-2*00501	FPFRRNFEFNL	0.25
14.	BoLA-4*02401	KGLNRLFPF	0.03
15.	”	RLFPFRRNFEF	0.08
16.	”	FEFNLTHSLLF	0.10
17.	”	RLFPFRRNF	0.40
18.	”	KRKPHSTSF	0.40
19.	”	FPFRRNFEF	0.50
20.	BoLA-5*03901	RLMNNLEFL	0.08
21.	”	RLMNNLEF	0.40
22.	BoLA-5*00301	RLMNNLEFL	0.03
23.	”	RLFPFRRNFEF	0.05
24.	”	RLMNNLEF	0.17
25.	”	RLFPFRRNF	0.30
26.	”	FMFGELLRL	0.40
27.	BoLA-6*01301	RLFPFRRNFEF	0.10
28.	”	RLFPFRRNF	0.17
29.	BoLA-6*04101	GELLRLMNNL	0.12
30.	”	FEFNLTHSL	0.17
31	”	FEFNLTHSLL	0.17
32.	”	LEFLNHEL	0.30

A rank threshold below or equal to 0.5 shows the peptide binds strongly with the corresponding BoLA type. A rank threshold above 0.5 but below 2 is considered a weak binding peptide. Only strong binding peptides are shown in this table.

**Fig 10:**
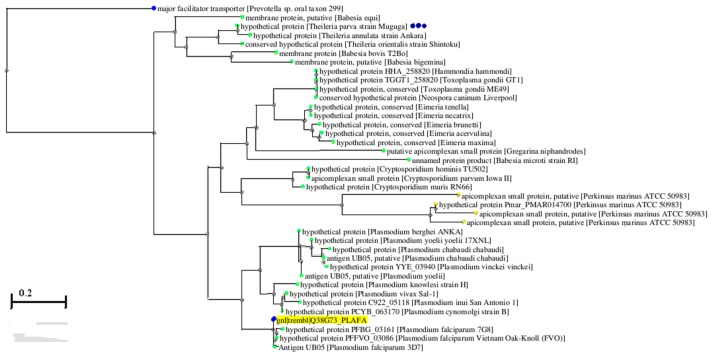
Phylogenetic analysis of TpUB05. Using algorithms at NCBI, a phylogenetic tree of TpUB05 was generated showing its relationship to the others found in apicomplexans.

The phylogenetic relationship between TpUB05 and its orthologues in apicomplexans was predicted using tools freely available at the NCBI. The analyses revealed a close ancestral relationship between TpUB05 and UB05 hence having a common ancestor Fig 10.

## Discussion

The present investigation shows for the first time that the antigen TpUB05 is an important marker of protective immunity against East Coast fever. This view is supported by the development of significantly higher antibody and T-cell responses in ITM vaccinated as compared to control animals, Further more, the observation that specific rabbit antibodies raised against r-TpUB05 were shown to block the invasion of cattle PBMCs by *T*. *parva in vitro*, further suggest that TpUB05 contributed to the protective immunity in the ITM-vaccinated cattle.

The titers of antibodies to TpUB05 were consistently higher in animals immunized against ECF than in naїve ones, even though in *in vitro* infection blocking assay, the antibody neutralizing activity was demonstrated with only 50% of the animals tested. The lack of complete inhibition of infection may be due to (i) a low level of expression of TpUB05 in the sporozoites as compared to the schizonts as revealed by RT-PCR or (ii) native TpUB05 may exist in a conformation that does not permit optimal binding with the anti-r-TpUB05 antiserum. A low transcriptional level of TpUB05 could also lead to its low copy number on the sporozoites surface that might have reduced the efficiency of the parasite neutralizing mechanism(s). Another explanation for this observation could be that the avidity and/or affinity of the antibody are weak. Also different animals might respond differently when inoculated with the same antigen [26].

IFN-γ has been shown to be an important cytokine of the Th-1 response, which plays an important role in the induction of protective immunity against many intracellular parasitic infections [27–29]. In our study, the TpUB05 antigen induced IFN-γ production as well as T-cell proliferation in 8 out of 12 ITM-immunized animals. This indicates that ITM vaccinated animals have TpUB05 memory T-cells that expand and produce cytokines upon re-stimulation with TpUB05. Hence, TpUB05 possesses T-cell epitopes that are presented by bovine antigen presenting cells in association with their corresponding restricting MHC class I. It is expected that only PBMCs from animals with the appropriate MHC class I haplotypes for presenting TpUB05 epitopes were stimulated to produce and release IFN-γ. The fact that animals of different MHC class I background were stimulated suggests the presence of either several epitopes or a promiscuous T-cell target epitope in TpUB05. Further analyses such as cytotoxic assay and epitopes characterization need to be done.

CD4+ T cells are known to play a crucial role in the induction of naive CD8+ T cell responses, it may therefore be important to include antigens that contain CD4+ helper T cell epitopes in a CTL-targeted vaccine.

The observed higher SI values from the T-cell proliferation as compared to the ELISpot assays could be explained by the targeting of whole PBMCs in the former and only CD4+ and CD8+ in the latter. Innate effector T-cells have been shown to respond to antigen without prior exposure. This subset of cells are primarily located in the thymus but are also found in circulation [27, 29] and also possess rapid effector function. One such innate effector, the invariant natural killer (NK) T cells (iNKT) does express a semi-variant T-cell receptor and can recognize “new” antigen and become activated to produce a variety of cytokines including IFN-γ [28, 29]. This could explain why one of the naive animals in this study produced IFN-γ significantly upon stimulation with TpUB05. This also strengthens the point that TpUB05 could as well be a potential marker of protective immunity for ECF.

The demonstrating of cross reactivity of antibody raised to the two antigens, TpUB05 and UB05 is consistent with the extensive sequence homology; 43.3% identity and 67% similarity observed the two antigens. Consistent expression of the TpUB05 in the life cycle stages of *T*. *parva* lents support to the view that a plays a key function in the parasite.

The present investigation shows for the first time that TpUB05 (GenBank accession number: KF875450), a homologue of a *P*. *falciparum* immunodominant antigen is strongly recognized by antibodies and PMBCs from cattle successfully vaccinated against East Coast fever (*T*. *parva* infection) using the infection followed by treatment (ITM) protocol. This implies that TpUB05 is involved in the immune response to cattle immunized against ECF. Our study also suggests the interesting possibilty that conserved homologuous antigens from phylogenetically related pathogens may be better stimulators of protective immunity. This is consistent with the strategy in vaccinology to use closely-related pathogens to create effective vaccines, for example, the cow pox virus to protect against small pox virus in the classical Jenner vaccine [30]; the successful use of *Mycobacterium bovis* to develop the well-known BCG vaccine to protect humans against *Mycobacterium tuberculosis* infection [31]. Also other investigators have shown that there is cross protection against *Onchocerca volvulus* infection by exposure to *O*. *ochengi* [32]. Thus, the potential role of TpUB05 in inducing immune protection against East Coast fever merits further investigation.

## Supporting Information

S1 Fig cDNA and predicted amino acid sequence of the TpUB5 from *Theileria parva*. TpUB05 amplicon was purified from agarose gel, cloned into pGEM-T Easy vector (Promega) then excised with BamHI and HindIII restriction enzymes digestion and subcloned into pET32a+ (Novagen) digested with BamHI and HindIII. The recombinant pET32/TpUB05 plasmid was sequenced using the vector T7 promoter and T7 terminator primers. The nucleotide sequence of the 300 bpTpUB05 amplicon shown is flanked by forward and reverse PCR primers regions double-underlined with the respective 5’ BamHI and 3’ HindIII directional cloning sites shadowed. The open reading frame starts with the nucleotide number 1 of the introduced BamHI site and ends with the 3’ TAA stop codon shown in bold italics. The first two amino acid residues of the 97 single letter peptide encoded by the cDNA correspond to the in-frame BamHI cloning site. The asterisk (*) indicates the stop codon.
(DOC)

S2 Fig Sequence of the TpUB05/pET32a+ fusion partner. (A) Nucleotide sequence of TpUB05 fusion protein cloned into pET32a+. The TpUB05 fusion protein is encoded by a 789 bp DNA fragment composed of the plasmid vector sequence underlined and the TpUB05 cDNA. The BamHI and HindIII used to clone the cDNA fragment are shown in bold italic letters. (B) The predicted amino acid sequence of the TpUB05 fusion protein. In the 260 amino acid single letter peptide derived from the recombinant TpUB05 cDNA, the vector portion is underlined and the 6x His tag used for affinity purification is shadowed.
(DOC)

S3 Fig Titration of anti-TpUB05 antiserum. 10 μg/ml r-TpUB05 was used to titrate the anti-TpUB05 antiserum using ELISA. This gave a titre of 1:128000.
(TIF)

S1 Table Intensity fraction. A: Using GelQuant.NET Version 1.8.2 (BiochemLAbSolutions.com), the intensity fractions, shows that, the expression of the house-keeping GAPDH is almost the same for all the stages while that of TpUB05 changes significantly between life cycle stages. B: Normalizing the intensity of TpUB05 with respect to GAPDH in the various major life cycle stages.
(DOC)

S2 Table ELISA using Tag-only antigen. Tag only was tested in ELISA against the serum of all test animals. The OD values obtained was comparable to that of the naïve cattle.
(DOC)

S3 Table Quantification of western blot signals. Using GelQuant.NET Version 1.8.2 (BiochemLAbSolutions.com), the intensity fractions of the bands of the western blot experiment was determined.
(DOC)
