# Supplementary material for: TpUB05, a Homologue of the Immunodominant Plasmodium falciparum Protein UB05, Is a Marker of Protective Immune Responses in Cattle Experimentally Vaccinated against East Coast Fever
Source: PLoS One. 2015 Jun 8;10(6):e0128040. doi: 10.1371/journal.pone.0128040 (PMC4459990; doi:10.1371/journal.pone.0128040)
Supplement: S1 Fig — TpUB05 amplicon was purified from agarose gel, cloned into pGEM-T Easy vector (Promega) then excised with BamHI and HindIII restriction enzymes digestion and subcloned into pET32a+ (Novagen) digested with BamHI and HindIII. The recombinant pET32/TpUB05 plasmid was sequenced using the vector T7 promoter and T7 terminator primers. The nucleotide sequence of the 300 bpTpUB05 amplicon shown is flanked by forward and reverse PCR primers regions double-underlined with the respective 5’ BamHI and 3’ HindIII directional cloning sites shadowed. The open reading frame starts with the nucleotide number 1 of the introduced BamHI site and ends with the 3’ TAA stop codon shown in bold italics. The first two amino acid residues of the 97 single letter peptide encoded by the cDNA correspond to the in-frame BamHI cloning site. The asterisk (*) indicates the stop codon. (DOC) [file pone.0128040.s001.doc]

**Supporting information: Molecular Cloning and expression of TpUB05**

Using *TpUB05* specific primers, a 300 bp DNA fragment encoding a 95 amino acid long protein of 11.2kDa (not including 2 amino acids from the in-frame 5’end BamHI restriction site) was amplified from schizont derived RNA (Fig. 1A) Subsequent cloning and expression in pET32a+ vector yielded a 28.9 KDa recombinant fusion protein as shown by SDS-PAGE. The increase in size to 260 amino acids was fully accounted for by the presence of 6xHis tag, as well as the S Tag, thrombin and enterokinase domains and a 109 amino acid thioredoxin fusion protein partner (S1 and S2 Figs).

1 ggatccgcagatctcaccaaacgcaaacctcactcaacttctttcgtcgatctcacacga 60

1 ***G S*** A D L T K R K P H S T S F V D L T R 20

61 ttcctagatagtggtgtgttgacattatttacggtgttgttgtcttgcacttttctattt 120

21 F L D S G V L T L F T V L L S C T F L F 40

121 atgtttggggagcttttgagactcatgaataacctcgaattcctcaaccacgaactcgtc 180

41 M F G E L L R L M N N L E F L N H E L V 60

181 aaaaaaggactcaatcgcctcttcccattccggagaaactttgagttcaatttgacgcat 240

61 K K G L N R L F P F R R N F E F N L T H 80

241 tctctactattttcagtatgcgttttacttcacagtttcaggagatcaaac***taa***aagctt 300

81 S L L F S V C V L L H S F R R S N * 97
