# Supplementary material for: TpUB05, a Homologue of the Immunodominant Plasmodium falciparum Protein UB05, Is a Marker of Protective Immune Responses in Cattle Experimentally Vaccinated against East Coast Fever
Source: PLoS One. 2015 Jun 8;10(6):e0128040. doi: 10.1371/journal.pone.0128040 (PMC4459990; doi:10.1371/journal.pone.0128040)
Supplement: S2 Fig — (A) Nucleotide sequence of TpUB05 fusion protein cloned into pET32a+. The TpUB05 fusion protein is encoded by a 789 bp DNA fragment composed of the plasmid vector sequence underlined and the TpUB05 cDNA. The BamHI and HindIII used to clone the cDNA fragment are shown in bold italic letters. (B) The predicted amino acid sequence of the TpUB05 fusion protein. In the 260 amino acid single letter peptide derived from the recombinant TpUB05 cDNA, the vector portion is underlined and the 6x His tag used for affinity purification is shadowed. (DOC) [file pone.0128040.s002.doc]

**A. nucleotide sequence**

ATGAGCGATAAAATTATTCACCTGACTGACGACAGTTTTGACACGGATGTACTCAAAGCGGACGGGGCGATCCTCGTCGATTTCTGGGCAGAGTGGTGCGGTCCGTGCAAAATGATCGCCCCGATTCTGGATGAAATCGCTGACGAATATCAGGGCAAACTGACCGTTGCAAAACTGAACATCGATCAAAACCCTGGCACTGCGCCGAAATATGGCATCCGTGGTATCCCGACTCTGCTGCTGTTCAAAAACGGTGAAGTGGCGGCAACCAAAGTGGGTGCACTGTCTAAAGGTCAGTTGAAAGAGTTCCTCGACGCTAACCTGGCCGGTTCTGGTTCTGGCCATATGCACCATCATCATCATCATTCTTCTGGTCTGGTGCCACGCGGTTCTGGTATGAAAGAAACCGCTGCTGCTAAATTCGAACGCCAGCACATGGACAGCCCAGATCTGGGTACCGACGACGACGACAAGGCCATGGCTGATATC***GGATCC***GCAGATCTCACCAAACGCAAACCTCACTCAACTTCTTTCGTCGATCTCACACGATTCCTAGATAGTGGTGTGTTGACATTATTTACGGTGTTGTTGTCTTGCACTTTTCTATTTATGTTTGGGGAGCTTTTGAGACTCATGAATAACCTCGAATTCCTCAACCACGAACTCGTCAAAAAAGGACTCAATCGCCTCTTCCCATTCCGGAGAAACTTTGAGTTCAATTTGACGCATTCTCTACTATTTTCAGTATGCGTTTTACTTCACAGTTTCAGGAGATCAAACTAA***AAGCTT***

**B. Amino acid sequence**

*MSDKIIHLTDDSFDTDVLKADGAILVDFWAEWCGPCKMIAPILDEIADEYQGKLTVAKLNIDQNPGTAPKYGIRGIPTLLLFKNGEVAATKVGALSKGQLKEFLDANLAGSGSGHM****HHHHHH****SSGLVPRGSGMKETAAAKFERQHMDSPDLGTDDDDKAMADIGS*ADLTKRKPHSTSFVDLTRFLDSGVLTLFTVLLSCTFLFMFGELLRLMNNLEFLNHELVKKGLNRLFPFRRNFEFNLTHSLLFSVCVLLHSFRRSN*
