# Supplementary figures and images for: TpUB05, a Homologue of the Immunodominant Plasmodium falciparum Protein UB05, Is a Marker of Protective Immune Responses in Cattle Experimentally Vaccinated against East Coast Fever
Source: PLoS One. 2015 Jun 8;10(6):e0128040. doi: 10.1371/journal.pone.0128040 (PMC4459990; doi:10.1371/journal.pone.0128040)

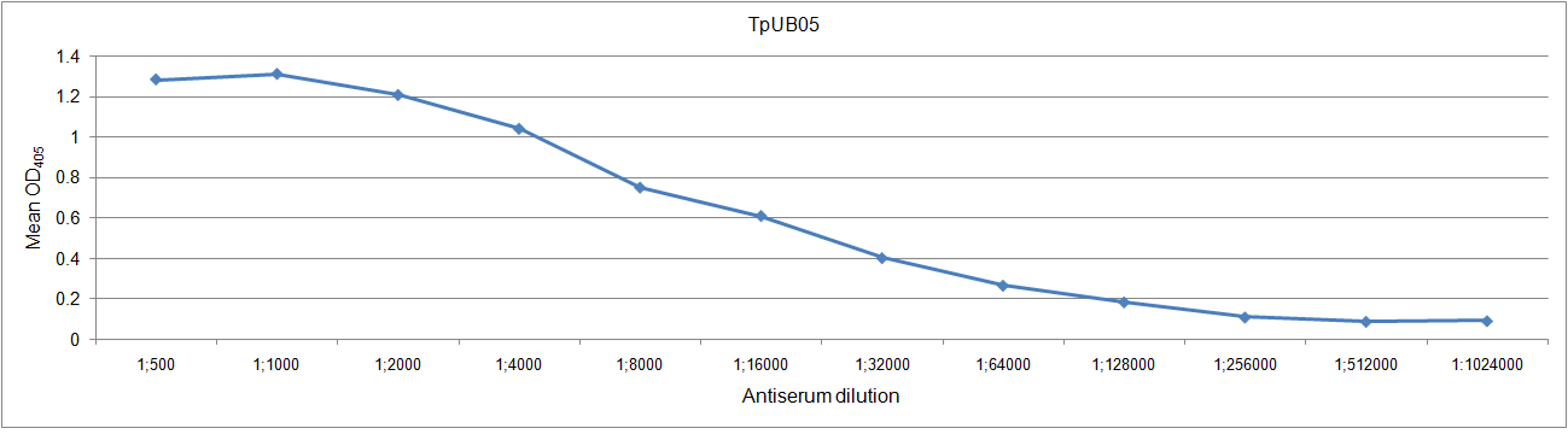

Supplement: S3 Fig — 10 μg/ml r-TpUB05 was used to titrate the anti-TpUB05 antiserum using ELISA. This gave a titre of 1:128000. (TIF) [file pone.0128040.s003.tif]
