# Supplementary material for: TpUB05, a Homologue of the Immunodominant Plasmodium falciparum Protein UB05, Is a Marker of Protective Immune Responses in Cattle Experimentally Vaccinated against East Coast Fever
Source: PLoS One. 2015 Jun 8;10(6):e0128040. doi: 10.1371/journal.pone.0128040 (PMC4459990; doi:10.1371/journal.pone.0128040)
Supplement: S1 Table — A: Using GelQuant.NET Version 1.8.2 (BiochemLAbSolutions.com), the intensity fractions, shows that, the expression of the house-keeping GAPDH is almost the same for all the stages while that of TpUB05 changes significantly between life cycle stages. B: Normalizing the intensity of TpUB05 with respect to GAPDH in the various major life cycle stages. (DOC) [file pone.0128040.s004.doc]

**S1 A Table**: Intensity fraction

|  | Sporozoite | Schizonts | Piroplasms |
| --- | --- | --- | --- |
| GAPDH | 0.30 | 0.34 | 0.36 |
| TpUB05 | 0.14 | 0.50 | 0.36 |

**S1 B Table**: Normalization of the intensity of TpUB05 with GAPDH

|  | Sporozoite | Schizonts | Piroplasms |
| --- | --- | --- | --- |
| GAPDH | 0.78 (100%) | 0.54(100%) | 0.64(100%) |
| TpUB05 | 0.22(28.3%) | 0.46(85%) | 0.36(56.3%) |
