# Supplementary material for: TpUB05, a Homologue of the Immunodominant Plasmodium falciparum Protein UB05, Is a Marker of Protective Immune Responses in Cattle Experimentally Vaccinated against East Coast Fever
Source: PLoS One. 2015 Jun 8;10(6):e0128040. doi: 10.1371/journal.pone.0128040 (PMC4459990; doi:10.1371/journal.pone.0128040)
Supplement: S2 Table — Tag only was tested in ELISA against the serum of all test animals. The OD values obtained was comparable to that of the naïve cattle. (DOC) [file pone.0128040.s005.doc]

|  | 1 | 2 | 3 | 4 | 5 | 6 | 7 | 8 |
| --- | --- | --- | --- | --- | --- | --- | --- | --- |
| animal no. | buffer alone  (no antigen) | **BD040** | **BD073** | **BD46** | **BD52** | **BC161** | **BD72** | **BC148** |
| Average OD | 0.028 | 0.041 | 0.074 | 0.048 | 0.059 | 0.066 | 0.051 | 0.053 |
| animal no. |  | **BD54** | **BD58** | **BC106A** | **BD028** | **BD028** | **BC143** | **BD88** |
| Average OD | 0.035 | 0.011 | 0.061 | 0.04 | 0.065 | 0.064 | 0.023 | 0.065 |
| animal no. |  | **BD61** | **BD63** | **BD69** | **BD81** | **BC150** | **BD80** | **BD76** |
| Average OD | 0.041 | 0.09 | 0.209 | 0.092 | 0.049 | 0.012 | 0.057 | 0.08 |
| animal no. |  | **BC138** | **BC110A** | **BC136** | **BD71** | **BD74** | **BD53** | **BC152** |
| Average OD | 0.033 | 0.04 | 0.068 | 0.071 | 0.067 | 0.089 | 0.081 | 0.055 |

**S2 Table: ELISA using Tag-only antigen**
