# Supplementary material for: TpUB05, a Homologue of the Immunodominant Plasmodium falciparum Protein UB05, Is a Marker of Protective Immune Responses in Cattle Experimentally Vaccinated against East Coast Fever
Source: PLoS One. 2015 Jun 8;10(6):e0128040. doi: 10.1371/journal.pone.0128040 (PMC4459990; doi:10.1371/journal.pone.0128040)
Supplement: S3 Table — Using GelQuant.NET Version 1.8.2 (BiochemLAbSolutions.com), the intensity fractions of the bands of the western blot experiment was determined. (DOC) [file pone.0128040.s006.doc]

S3 Table: Quantification of western blot signals.

|  | lane 2: anti-TpUB05 antiserum; | lane 3: antiserum from ITM-immunized cattle BD053 | lane 4: antiserum from un-immunized infected but not treated cattle BD040; | lane 5: anti-UB05 antiserum |
| --- | --- | --- | --- | --- |
| Intensity (intensity fraction) | 59348.5  (0.32) | 42805.7  (0.24) | 3072.9  (0.01) | 80604.9  (0.43) |
